# Factors influencing referral to maternity models of care in Australian general practice

**DOI:** 10.1371/journal.pone.0296537

**Published:** 2024-05-21

**Authors:** Jaime Thomas, Lesley Kuliukas, Jacqueline Frayne, Zoe Bradfield

**Affiliations:** 1 School of Nursing, Curtin University, Bentley, Western Australia, Australia; 2 Medical School, University of Western Australia, Albany, Western Australia, Australia; Deakin University Faculty of Health, AUSTRALIA

## Abstract

**Background:**

In the Australian maternity system, general practitioners play a vital role in advising and directing prospective parents to maternity models of care. Optimising model of care discussions and the decision-making process avoids misaligning women with over or under specialised care, reduces the potential for disruptive care transitions and unnecessary healthcare costs, and is critical in ensuring consumer satisfaction. Current literature overwhelmingly focusses on women’s decision-making around model of care discussions and neglects the gatekeeping role of the General Practitioner (GP). This study aimed to explore and describe the factors influencing Australian GPs decision-making when referring pregnant women to maternity models of care.

**Methods:**

This study used a qualitative descriptive approach. General practitioners (N = 12) with experience referring women to maternity models of care in Australia participated in a semi-structured interview. Interviews occurred between October and November 2021 by telephone or videoconference. Reflexive thematic analysis was facilitated by NVivo-12 data management software to codify and interpret themes from the data.

**Findings:**

Two broad themes were interpreted from the data. The first theme entitled ‘GP Factors’, incorporated three associated sub-themes including ‘1) GPs Previous Model of Care Experience’, ‘2) Gaps in GP Knowledge’ and ‘3) GP Perception of Models of Care’. The second theme, entitled ‘Woman’s Factors’, encapsulated two associated sub-themes including the ‘4) Woman’s Preferences’ and ‘5) Access to Models’.

**Conclusions:**

This study provides novel evidence regarding general practitioner perspectives of the factors influencing model of care decision-making and referral. Predominant findings suggest that gaps in GP knowledge regarding the available models of care are present and are largely informed by prior personal and professional experience. Most GPs described referring to models of care they perceive positively and centring their model of care discussions on the woman’s preferences and accessibility. The exploration and description of factors influencing model of care decisions provide unique insight into the ways that all stakeholders can experience access to a broader range of models of care including midwifery-led continuity of care models aligned with consumer-demand. In addition, the role of national primary health networks is outlined as a means to achieving this.

## Introduction

The Australian healthcare system and its maternity services operate through a two-tier system of both public and private providers [1, 2]. The national publicly funded universal healthcare program, Medicare, provides hospital and community-based care to Australian and New Zealand citizens [2, 3]. Consumers with Medicare accessing public hospital care predominantly experience no out-of-pocket costs, while community-based care is made more affordable through the provision of government-funded rebates [2, 3]. Conversely, private hospitals and services are accessible with private health insurance and a co-payment or alternatively, incur a full fee for service [2, 3].

Within this system, almost all pregnant women will make initial contact with a general practitioner (GP) when commencing their maternity care journey [4, 5]. GPs provide healthcare across the lifespan and play a key role in advising and directing prospective parents to a maternity model of care [2]. There are many factors that determine birth location and care provider for women. Beyond maternal choice, factors such as geographic location, visa status, medical conditions and socioeconomic identity may impact the models of care available [1, 6]. In addition, GPs may consider the service delivery context, the patient’s health insurance status and the available expertise prior to making a referral [7–9]. They have a gatekeeping function in the way that access to certain models of care is only granted through the GP referral [2]. For example, in Australia, access to a private midwifery service requires a ’collaborative arrangement’ between the participating midwife and medical practitioner in order for the services to be subsidized by Medicare [10]. Similarly, a GP referral is required when accessing private obstetric models of care in order for women to receive a Medicare rebate [2, 11]. It is also important to note that GPs working in the GP obstetrician model of care can be both referring provider and the maternity care provider [2, 12]. Although other referral pathways exist such as through other clinicians or via self-referral, the GP referral pathway is the most common way women access models of care [2, 13].

In Australia, there are 11 models of maternity care according to the Maternity Care Classification System (MaCCs), each has their own specific features, personnel, and fee structure [2, 12]. These can be grouped into five major model of care categories which include standard public care, shared care, public midwifery continuity care, private obstetric care and private midwifery care [2, 12]. Standard public care is provided by hospital doctors and midwives and is government funded [2, 12]. Shared care involves antenatal care delivered by a community doctor and or midwife, with several key antenatal visits and intrapartum care occurring in the public hospital [2, 12]. Hospital-based care within a shared care model is government funded though women may incur out of pocket costs for GP-led visits [2, 12]. Public midwifery continuity of care models are government funded and provided by a hospital midwife or small team of midwives with birth largely occurring in a birth centre or public hospital [2, 12]. Within the public midwifery continuity of care category is midwifery group practice caseload care. Midwifery group practice caseload care involves antenatal care provided by a known midwife or group of midwives in the hospital, community, home, or birth centre setting with intrapartum care taking place in the hospital, birth centre or home [2, 12]. Private obstetric care is provided by an obstetrician with birth occurring in a private or public hospital and is largely accessible via women’s private health insurance though some Medicare rebates may be available [2, 11, 12]. Subsumed within the private obstetric care major model category is general practitioner obstetrician care which involves community or hospital clinic antenatal care and public or private hospital intrapartum care provided by a GP obstetrician [2, 12]. Finally, private midwifery care involves care by a privately practising midwife who supports birth in the home or hospital settings and can be partly government funded for antenatal, postnatal and hospital intrapartum care and partly consumer funded [2, 12].

It is known that 97% of Australian women intend to and will give birth in a hospital setting but little is known about how women distribute across the models of care at a national level as this is not yet routinely reported [2, 14]. The first national model of maternity care report was published in November 2021, has been re-released annually and focusses on the maternity model of care characteristics [1, 14, 15]. As such, there remains limited data comparing and evaluating the models of care in Australia [2, 8, 16, 17]. Despite this, model of care report data and birthplace data can be used to infer how women distribute across the models of care. In 2023, approximately 1000 model of care services were active in Australia and most of these identified as public hospital maternity care (41%), 14% were midwifery group caseload care models, 15% identified as shared care models and 11% were private obstetrician specialist care models [14]. In 2019, Australian birthplace data revealed that 75% of women gave birth in a public hospital, 22% in a private hospital, 2.3% in a birth centre, 0.6% of women constituted births before the arrival to a health service and 0.3% of births occurred in the home setting [18].

### Significance

In 2009, the Australian Department of Health and Ageing published a maternity service review and recommended that there be improved model of care choice for women, a greater range of models of care be made available, an expanded role for midwives, improved access for Indigenous and rural women and further development of collaborative models of care [19]. This was strengthened by the most recent national maternity Strategy built on four values of choice, safety, access and respect orientated by woman-centred care [20].

Model of care information, when comprehensive, allows parents to make trade-offs between the perceived advantages and disadvantages and select a framework that most aligns with their preferences [8, 21]. In addition, model of care decision making when optimised, enhances consumer satisfaction, prevents over or under specialised care, prevents excessive healthcare costs and the potential for disruptive care transitions [2, 22].

The Fourth National Atlas in Healthcare Variation which identifies potentially unwarranted variation in healthcare has highlighted early planned birth by caesarean section and induction of labour before 39 weeks without medical indication as an area of concern [23]. It is well accepted that midwifery-led continuity of care confers a range of benefits to mothers and infants including a higher chance of a normal labour and birth and less chance of intrapartum caesarean section [24, 25]. Moreover, midwifery continuity of care is the only health system intervention shown to reduce preterm birth and improve perinatal survival [24, 25]. Despite the range of consumer and health system benefits evident among midwifery-led continuity of care models, relatively few Australian women have access to these models and it is the GP referral that often determines model of care and birthplace [26].

Models of care are also vital for the provision of culturally sensitive care for Aboriginal and Torres Strait Islander women and those from culturally and linguistically diverse (CALD) backgrounds. Midwifery-led continuity of care models that are tailored to the needs of Aboriginal and Torres Strait Islander women, particularly those that offer ‘birthing on country’, have been shown to improve health outcomes and confer a range of benefits to consumers [27–31]. Improvements associated with these models include high levels of consumer satisfaction [28–30], improved antenatal attendance rates [31], improved breastfeeding rates [31], and a reduction in preterm birth rates [28, 32]. For CALD women, there are a range of service elements such as continuity of care, effective communication, culturally responsive care, support navigating systems and flexibility within services that align with maternal preferences, reduce barriers to access and have the potential to improve outcomes for CALD mothers and infants [33].

It is recognised that challenges navigating the referral system can act as a barrier to initiating antenatal care which is vital for the health and wellbeing of women and babies [34]. Finally, the literature overwhelmingly focusses on women’s decision-making and neglects the gatekeeping role of the GP [35–37]. Given the established importance of GP referral to model of care and dearth of evidence regarding factors that influence referral, this study aimed to explore and describe the factors influencing Australian GPs’ decision-making when referring pregnant women to maternity models of care.

## Methods

A qualitative description design was used given the recognised utility to derive rich descriptions from participants and its suitability for understanding novel phenomena/on [38, 39]. The research aim prompted the use of a critical realist lens which is ontologically realist asserting that an authentic reality exists separately to human knowing while also being epistemologically subjectivist asserting that multiple versions of reality can be produced [40]. The critical realist lens informed the use of semi-structured interviews for data collection so as to appreciate the multiple versions of reality detailed by GPs. Moreover, its alignment with the reflexive thematic analysis process allowed for the development of key themes from the data thus fulfilling the assumption that an authentic reality exists separately to human knowing.

### Sampling and recruitment

A purposive, snowball and convenience sampling approach was taken utilising social media and email to recruit general practitioners from around the nation experienced in referring pregnant women to maternity models of care. Inclusion criteria sought to recruit participants working as GPs in Australia with experience referring pregnant women to maternity models of care. GPs from across all geographical locations were eligible to participate in this study including those from metropolitan, rural and remote areas. To work unsupervised as a Specialist Vocationally Registered GP in Australia, doctors undergo the Australian General Practitioner Training (AGPT) Program [41]. Before applying or completing the AGPT, doctors may work in general practice contexts and gain maternity referral experience. As such, all doctors working clinically in the general practice setting, those both vocationally registered and those not registered were included in the study. Doctors working outside Australia, without maternity referral experience were excluded. Recruitment occurred from the 23^rd^ of August 2021 to the 20^th^ of October 2021 with a conservative aim to recruit between 10 and 15 GPs given the time poor nature GPs. Prospective participants were provided with information on the study, the researchers involved, and its purpose via email; written consent was gained. An email reminder was provided to all participants 24 hours prior to interview.

### Data collection

GPs participated in a semi-structured interview conducted by researcher JT between October and November of 2021. From the recruitment period to interviews only one GP was lost from the study due to multiple scheduling conflicts. Sampling was driven by data saturation and conceptualised per Braun and Clarke’s recommendation [42], as the point at which limited new concepts or descriptions directly relevant to the research aim could be interpreted. Twelve general practitioners comprised the final sample size. Interviews were conducted via Microsoft Teams or via telephone and lasted between 19 and 33 minutes. Demographic data were collected at the beginning of the interview. A semi-structured interview guide was used (S1 Appendix) to explore the factors that influence GPs’ model of care decision making. It was developed from the research question and structured so as to provide an open-ended initial question with several listed prompts to be used as required by researcher JT. GP participants were initially asked “*Please describe how you decide where and to whom you refer your maternity clients*?” and were prompted as required. Interviews were audio recorded and transcribed verbatim by researcher JT. Participant details were de-identified and replaced with a participant number (e.g., P1) so as to maintain confidentiality.

### Data analysis

Interview transcripts then underwent Braun and Clarke’s reflexive thematic analysis facilitated by NVivo-12 data management software including 1) familiarisation with the data, 2) coding the data, 3) identifying themes, 4) reviewing themes, 5) defining and naming themes, and 6) producing the report [43]. To enhance the rigor of the study, the researcher (JT) maintained an audit trail and engaged in peer-review at relevant intervals with the research team to expose bias or inappropriate subjectivity. Approximately five meetings occurred over the course of the data analysis period which allowed the researcher to complete a ‘sense-check’ with the research team so as to enhance the richness of the interpretation [44].

The data were managed utilising an encrypted, password protected cloud storage platform (OneDrive) and university research drive. Following the interviews, data copies were deleted from OneDrive and will remain on the university research drive for a period of seven years.

This study obtained ethical clearance from the Curtin University Human Research Ethics Committee in alignment with the National Health and Medical Research Council guidelines (HREC 2021–0500).

## Findings

The demographic profile of the participants is outlined in Table 1. Of the twelve participants, eleven identified as female. Participants were between 30 and 59 years of age with most participants aged between 40 and 49 years. Most participants were GP Fellows of the Royal Australian and New Zealand College of General Practitioners. Five participants had less than 10 years of experience working in general practice, five participants had between 11 and 20 years of experience and two participants had more than 20 years of experience working in general practice. Most participants were currently living and working in Western Australia. Most of the participating GPs were working in metropolitan areas however five participants were currently working in a rural or regional area.

**Table 1:** Demographic profile of participants.

Demographic variables	Participant numbers n = 12
**Gender** Female Male	11 1
**Age** 30 to 39 40 to 49 50 to 59	4 6 2
**Level of vocation** GP Registrar Fellow	1 11
**Years of experience as a GP** 0 to 10 11 to 20 21 to 30	5 5 2
**Additional obstetric qualification** Nil Basic Diploma Advanced Diploma	5 6 1
**Maternity model work experience** Nil Birth Centre GP Obstetric Private hybrid model Hospital	2 3 7 1 10
**Years of experience in maternity model** Nil < 1 year 1 to 5 years 6 to 10 years Over 10 years	2 4 3 1 2
**Geographical classification** Metropolitan Rural Regional	7 4 1
**State** WA NSW QLD	8 3 1

**Fig 1:**
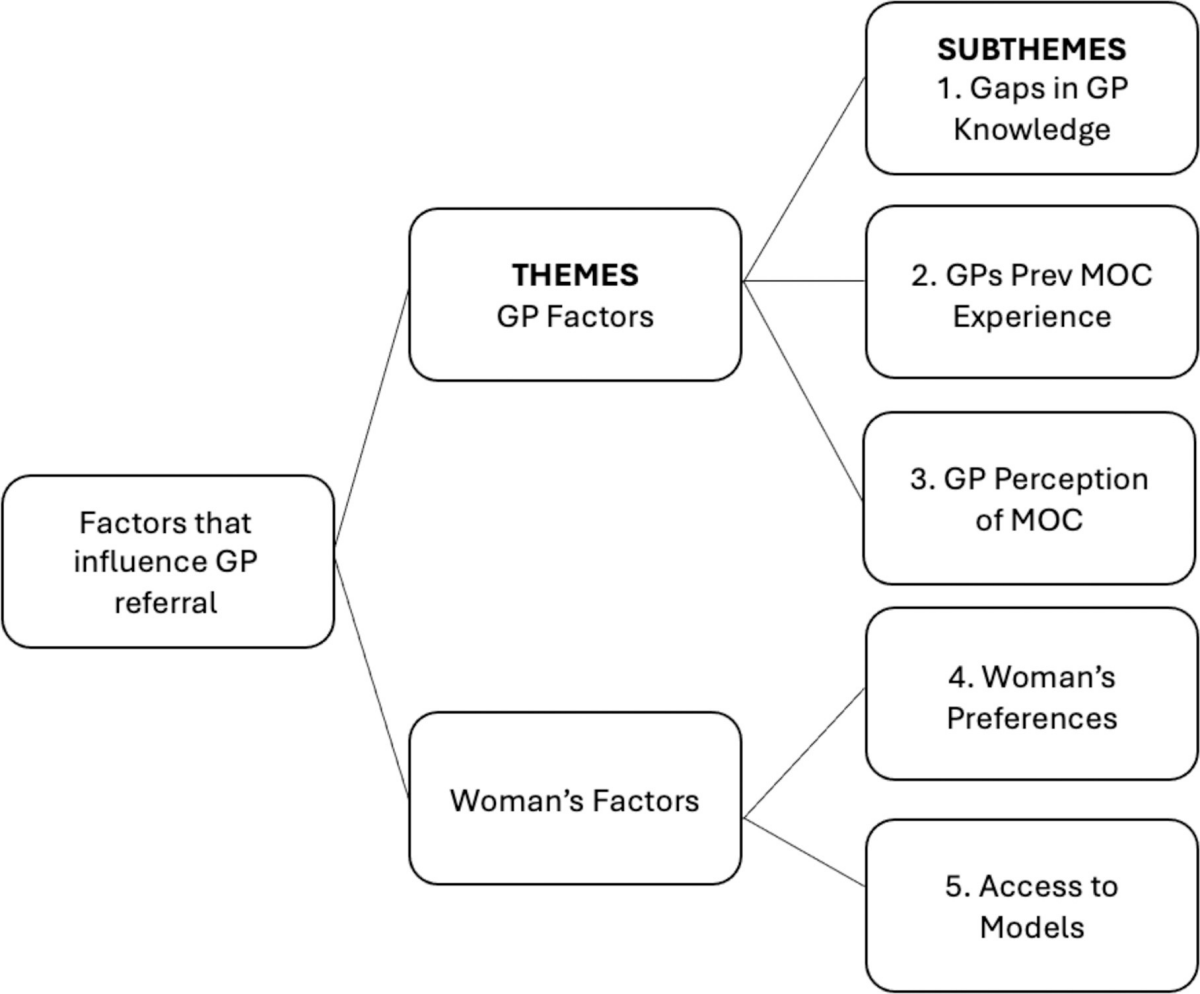
Final thematic map of the factors that influence GP referral to maternity models of care demonstrating two themes and five subthemes.

Two main themes were generated from the data analysis process. The first theme, entitled “GP factors” incorporates three subthemes including “1) Gaps in GP knowledge”, “2) GPs previous model of care experience” and “3) GPs perception of the models of care”. The second theme or the “Woman’s factors” encapsulates two subthemes including “4) Woman’s preferences” and “5) Access to models” (Fig 1).

### GP factors

#### 1) Gaps in GP knowledge

The participant GPs were asked about the models of care they had previously referred to and those they considered themselves to be familiar with. Overall participants had incomplete knowledge of the Australian maternity models of care and limited insight into the gaps in their knowledge base. One participant stated:

*Well*, *I either go public or private because that’s all the options we have…” (P5)*

Most GPs described gaining their model of care knowledge from their work experience which predominantly occurred in a hospital setting. One GP participant said:

*It [was] just seeing women come through the birth suite and where they’ve come from and what their journey has been and figuring out from there if it was a model or what the model was… (P7)*

The GPs were most familiar with the medical models and were less knowledgeable about midwifery-led models of care:

*I feel like I know enough about the medical model[s] because they’re the ones that I’ve worked most in*, *but I feel like midwifery group practice*, *community midwifery programs*, *family birth centres we don’t get a lot of information about…” (P7)*

Of those interviewed, two GP participants mentioned their Primary Health Network (PHN) when describing their model of care education and resources. Participant 3 stated that they use the PHN developed *HealthPathways* web-based portal that supports clinicians’ referrals:

…*looking at the HealthPathways and seeing what’s actually available in my area when and I sort of do that at point of care so when a woman who comes in and requesting a referral for pregnancy I kind of open that up… (P3)*

#### 2) GP’s previous model of care experience

Previous model of care experience both personally and professionally was described as influential to the GP’s referral practice. In terms of personal birthing experience most GPs accessed private obstetric-led care. One GP said:

*I had my first baby in the private sector*, *I didn’t think things went particularly well*. *I had my second baby as a private patient in the public sector and things went exceptionally well so I was quite impressed with the public system so that of course has influenced me (P5)*

All GPs in the cohort had professional exposure to hospital-based models of care with fewer participants describing GP obstetrics and birth centre experience. One participant said:

*[Other GPs] might not be aware of other models of care like caseload midwifery if they haven’t had that sort of exposure (P10)*

Communication from a service was also seen as influential to the GP referral:

…*if a service is good at communicating with me I’m more likely to refer to it as well…if that service sends me a letter with good information or lets me know if something significant has happened*, *then I’m more likely to want to continue to use that service (P6)*

#### 3) GP’s perception of the models of care

When asked about their rationale for making previous referrals to the maternity models of care, GPs described referring to models that they perceive positively one participant said:

*Well*, *I’m happy with all of them (P4***)**

GPs described referring to providers that they had confidence, faith, and trust in for example one GP stated:

**…** *the most common model of care that I refer to would be delivering with general practitioners in a [regional centre] and I think that’s because I have confidence in the service (P12)*

Positive word of mouth from women was described by most GPs as a factor that increased their referrals to a service or provider stating …*you get good reports about somebody you tend to refer to them more (P12)*

On the other hand GPs said that negative feedback from women about their model of care made them less likely to make future referrals to that service.

Most interviewed GPs had an unfavourable perception of private midwifery models of care offering homebirth, one participant stated:

*I generally don’t refer [to] that model of care ‘cause I guess …I’m more worried about their backup systems when things go wrong (P12)*

### Woman’s factors

#### 4) Woman’s preferences

When GPs were asked about how they make their referral decisions, they overwhelmingly indicated, *…so the predominant way is through patient choice… (P10)*. This included considering the woman’s desired mode of birth or birth location.

One GP said:

**…** *just talking through the pros and cons of each model*, *they may lean strongly towards one so they may have a preference for more midwife-led care or a preference for avoiding intervention if possible or …they’re just very keen on caesar or induction early on in the pregnancy that may influence how we make a decision…(P6)*

A patient or woman-centred philosophy was emphasised by the GPs explaining:

**…***if you follow what they want*, *they tend to end up with a better outcome*, *sort of more happy patients and… [if] a patient is contented with their pregnancy and delivery*, *is more likely to be a contented mother… (P5)*

Even when questioned about their feelings towards a woman declining a referral, GPs maintained:

**…***I’d feel fine I’d respect that…she has complete capacity to make that decision about her and her baby so that would be fine…her birth her pregnancy*, *her choice really yeah (P11)*

#### 5) Access to models

Access around the models of care was described by GPs as a major consideration during referral discussions.

Rural and regional GPs highlighted how women living in these areas are limited to a few models of care explaining:

*…there’s …limited options ‘cause we live in the country…- the options are what they are… (P12)*

The maternal and fetal risk profile was described as a directing force during referral discussions. It was often referenced as a contraindication to the woman’s choice. One GP said:

**…** *you know it’s their pregnancy and sometimes that doesn’t work out you know they might actually have a high-risk pregnancy and they don’t get to do what they would like to do… (P2)*

Financial and health insurance status of the woman was also highly relevant to referral discussions. GPs described discussions around out of pocket costs for women with one GP saying:

.. *if they see me for GP shared care ’cause I’m in a private billing practice*, *so there will be out of pocket costs associated with every appointment that they come in to see me … when it comes to the midwifery-led models of care I sort of yeah explain what that means in terms of cost which are minimal (P1)*

## Discussion

To our knowledge, this is the first exploratory study to describe factors that influence GP referral to maternity models of care. Gaps in knowledge identified in this study may impact GPs ability to offer a comprehensive range of models to women. GPs descriptions of reduced knowledge of midwifery-led continuity of care models may mean that the benefits associated with these models such as reduced intervention and increased spontaneous vaginal birth [45], go unrealized. Given the important referring function of the GP it is likely that these referral practices may be impacting maternal and neonatal outcomes at a population health level.

Resources supplied by Primary Health Networks (PHNs) via the *HealthPathways* portal was described as being used by two GP participants during their interviews. PHNs can be an important source of information for GP knowledge of the maternity models of care. PHNs assist with health service planning so as to ensure that primary care meets the community’s needs. PHN-developed resources, such as the *HealthPathways* portal is used to provide local clinical management pathways and referral advice [46]. However, the portal is limited by its completeness and relevance of information across Australia. For example, the Western Australian *HealthPathways* portal does not include locally relevant model of care referral information and instead has adapted its ‘Antenatal Consult–First’ pathway from New Zealand [47]. Embedding a decision tool into the *HealthPathways* portal with the up to date and locally relevant model of care service information could provide GPs with support during model of care discussions and provide women with a broader range of model of care options.

Of the interviewed GPs, most participants described personal and work experience in the medical-led models of care and reported a heightened familiarity with those models. With most medical education and GP obstetric training occurring in medical -led- models, this medical focus appeared to impact the exposure and familiarity of GPs with non-medical led models. Moreover, GPs described referring to providers they had previously worked with, and when predominantly occurring in the hospital setting, this reinforced referral to medical providers. Whilst there are no studies that have previously explored medical referring within the maternity context, a cross-sectional study of American primary care physicians (n = 1252) demonstrated that together with other factors, past experience with a specialist was highly important to their referral practice [48]. Further, a prospective cohort study also conducted in the United States found that personal knowledge of a specialist was the most important consideration for primary care doctors when referring patients [49]. Given that most of the interviewed participants had hospital-based model of care experience, early indications are that GPs may preferentially choose obstetricians working in those models of care as opposed to community-based GP obstetricians or midwives.

GPs disclosed referring to providers they perceive positively. A study by Choudhry et al. found that broadly, referral recommendations made by physicians have little or no objective basis. Rather, that the physicians’ perception of the specialist’s reputation, knowledge base and feedback from previously referred patients instead are highly valued when making referral recommendations [50]. Addressing this practice is made difficult as one needs exposure to a model to develop a positive perception of that model. GP training that includes exposure to a variety of maternity models of care, including midwifery-led models of care, may broaden GPs’ perceptions of a range of models of care.

The woman’s choice and preferences were reported by GPs as central to the referral decision, giving insight into what they consider women view as important including birth location, mode of birth and continuity of carer. At face value, these findings may indicate GP alignment with the key intentions of the national maternity Strategy, particularly regarding choice and informed-decision making [20]. The reluctance demonstrated by some participants to refer to privately practising midwives in this research has been explored in previous studies [8, 51, 52]. In a national survey of n = 1657 participants, narratives from women describing challenges of having to ‘fight’ with their GP to obtain a referral to a private midwife or to receive midwife only care [52]. GP reluctance or refusal to refer to midwifery models of care requires further exploration at a national policy level. Changes in the National Law requiring private midwives to have a collaborative arrangement with a GP are underway, it is anticipated that this will improve women’s access to private midwifery and remove the requirement for referral from a GP [10].

Finally, GPs described access to the models of care as a key consideration during interviews. GPs reported how rural and remote women have limited options for maternity care. This finding is congruent with current literature that outlines how women residing in remote geographical areas experience fewer or minimal options for maternity care access compared with their metropolitan counterparts [53]. Improving maternity care access for rural and remote women is a key objective within the national maternity Strategy. Strategies such as optimising the of the maternity workforce, service innovations such as telehealth and outreach care, as well as national pathways, are critical to achieving improved access for rural and remote women [20]. A national review of health professionals’ scope of practice is currently being undertaken by the Commonwealth government which aims to address barriers in equity and access for all Australians including women seeking rural maternity services [54]. The interviewed GPs also described how characteristics like maternal fetal risk profile, the financial status of the woman, and convenience, may mean that current model of care options do not meet the needs of women. Despite current risk stratification practices, there is compelling evidence regarding the benefits of continuity of midwifery care for women of all risk profiles alongside a multidisciplinary team [25, 55]. Although publicly funded models of care represent those most commonly accessed by Australian women, rising out-of-pocket costs serve as a barrier to accessing care particularly for those living in rural areas with limited primary maternity services offering Medicare rebates that cover the full cost of care [56]. Strategies such as funding models that support continuity of care for all women, shifting GP practice funding to support patients in need, bundled funding models, and expanding access to expert primary care through clinically activated PHNs have been discussed [20, 57–59]. The interviewed GPs described the woman’s proximity to the model of care as similarly important to other doctors when undertaking a referral [48, 50, 60]. The benefit of convenient, proximal maternity models of care include reduced work and family disruption, reduced travel related safety issues, and the ability to facilitate birthing on country, a priority for many Aboriginal women [20].

Maintaining currency of knowledge of the maternity models available within a local referring area presents a challenge for GPs that could be met by providing online resources to support broader conversations with women and referral practices. Around the country, individual jurisdictions have begun to address the gap in knowledge, examples include the Pregnancy, Birth and Baby website and the MyBaby WA App which targets new or expectant mothers but may also be used by health professionals to support a range of conversations including model of care choice [61, 62]. Education, beginning early on in medical schools, and in GPs training programs on the breadth of model of care options available is important to increase awareness and enable GPs to refer from a broader knowledge base. In their public health education role, GPs could partner with women to enable a shared understanding of their options so all stakeholders can experience the benefits of consumer-demand and led access to a wider variety of models.

Within GP education and training, exposure to non-hospital-based models of care may provide the opportunity for GPs and training doctors to gain a sense of familiarity and enhance their knowledge of those models. This and expanding the role of midwives in the primary health sector including in primary health network (PHN) stakeholder groups would foster networks between GPs and other providers improving interdisciplinary care and health service integration. Despite no current evidence evaluating the benefit of midwifery integration into primary care clinics or PHN stakeholder groups in Australia, a predominantly European study that surveyed GPs and patients across 34 countries evaluated GP outcomes and patient experiences of GPs co-located with other health professionals including midwives [63]. Of the 7183 GPs surveyed, most were co-located with nursing staff while only one in three described themselves as co-located with midwives, specialists, physiotherapists, dentists or pharmacists [63]. The analysis demonstrated that co-location with the aforementioned allied health staff or midwives resulted in improved coordination with secondary care [63]. As such, the integration of midwives into GP practices or other primary settings such as community centres may confer improved coordination with secondary care. More research into the benefits of midwifery integration into the primary health sector and in PHN stakeholder groups is needed.

Finally, insights gained around women’s ability to access models of care is helpful for health system planning with a view to improve health outcomes, choice for women, and the range of models of care available to them. Strengthening the rural workforce by training GP obstetricians, enabling scope fulfilment for midwives who have recognized expertise in primary maternity care, using telehealth and outreach services and national pathway development have been highlighted as vital to improving healthcare delivery to rural women [20, 64]. Moreover, the development of all-risk caseload midwifery models of care [25, 55], funding that supports continuity of care options [20, 57, 58] and model of care design that improves the convenience elements of maternity models [20] have the potential to better meet the needs of new and expectant parents in Australia.

## Strengths and weaknesses

The strengths of this study lie in the qualitative descriptive approach to this newly investigated topic. Selecting this methodology enabled an open exploration of the phenomena which provides novel evidence and delivers recommendations for further related areas of enquiry. Many of the participant GPs were from one Australian jurisdiction which may limit the generalisability of this research although this not a goal of qualitative research. The detailed demographic data provided enables the reader to determine the transferability of the study findings to their individual setting.

## Conclusion

Study findings have delivered novel evidence regarding the factors that influence GP referral to maternity models of care in Australia. There are known perinatal health, economic, societal benefits conferred at a population level for women who access selected models of care. The findings of this study provide important signposts about why and how models of care referral practices could be broadened and improved to access these conferred benefits for all Australian families. Further research, education and policy reform is recommended to improve women’s access to models of care via more informed GP referral practices.

## Supporting information

S1 Checklist Human participants research checklist. (DOCX)

S1 Appendix (TIF)
