# Supplementary figures and images for: Factors influencing referral to maternity models of care in Australian general practice
Source: PLoS One. 2024 May 21;19(5):e0296537. doi: 10.1371/journal.pone.0296537 (PMC11108194; doi:10.1371/journal.pone.0296537)

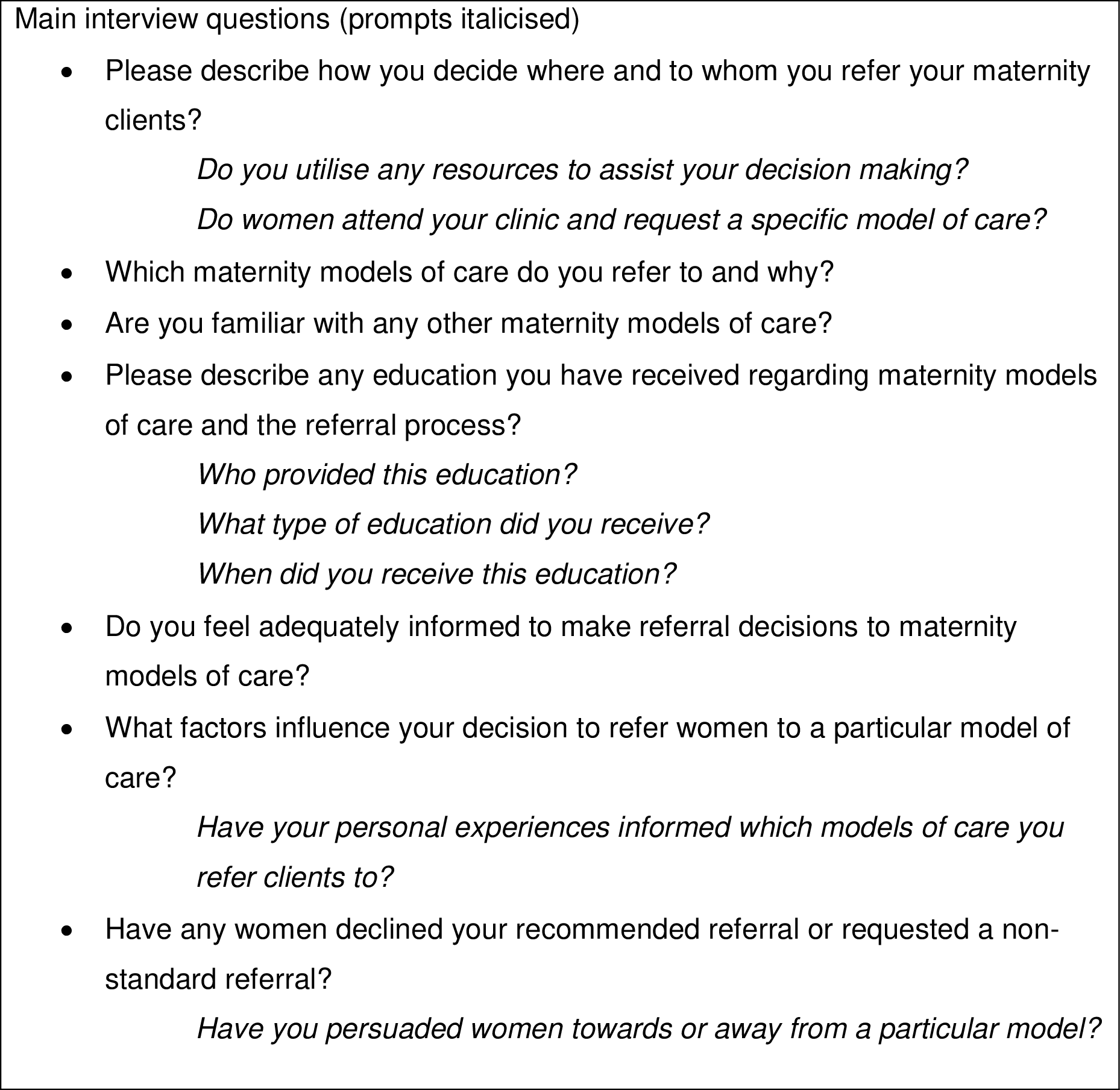

Supplement: S1 Appendix — (TIF) [file pone.0296537.s002.tif]
